# Correction to “Complete Mitochondrial Genomes of Eleven Extinct or Possibly Extinct Bird Species”

**DOI:** 10.1111/1755-0998.70181

**Published:** 2026-08-01

Anmarkrud, J. A. and J. T. Lifjeld . 2017. “Complete mitochondrial genomes of eleven extinct or possibly extinct bird species.” Molecular Ecology Resources 17, no. 2: 334–341.27654125 10.1111/1755-0998.12600

One of the sequenced specimens (Accession number: NHMO‐BI‐67809, Natural History Museum, University of Oslo) was wrongly identified as *Akialoa obscura*. The error was discovered during recent examination of the voucher specimen and its accession history. The correct species identification is *Akialoa stejnegeri*, an extinct species from the island of Kauai in Hawaii Islands, USA. The voucher specimen was part of one of two shipments of Hawaiian birds sent from a Norwegian collector, Valdemar Knudsen, in 1890 and 1893. Mr. Knudsen was a resident of Kauai Island.

To the best of our knowledge, this misidentification affects only the taxonomic assignment of that particular mitogenome and does not alter the sequence data, analytical procedures or the main conclusions of the study.

We thank Roland van der Vliet, Justin Jansen and Lars Erik Johannessen for bringing this misidentification to our attention.

We apologize for this error.

